# Gaussian of Differences: A Simple and Efficient General Image Fusion Method

**DOI:** 10.3390/e25081215

**Published:** 2023-08-15

**Authors:** Rifat Kurban

**Affiliations:** Department of Computer Engineering, Abdullah Gul University, 38080 Kayseri, Turkey; rifat.kurban@agu.edu.tr; Tel.: +90-(352)-224-88-00

**Keywords:** general image fusion, Gaussian of differences, multi-focus image fusion, medical image fusion, infrared and visible image fusion, multi-exposure image fusion, image quality metrics, pattern search optimization

## Abstract

The separate analysis of images obtained from a single source using different camera settings or spectral bands, whether from one or more than one sensor, is quite difficult. To solve this problem, a single image containing all of the distinctive pieces of information in each source image is generally created by combining the images, a process called image fusion. In this paper, a simple and efficient, pixel-based image fusion method is proposed that relies on weighting the edge information associated with each pixel of all of the source images proportional to the distance from their neighbors by employing a Gaussian filter. The proposed method, Gaussian of differences (GD), was evaluated using multi-modal medical images, multi-sensor visible and infrared images, multi-focus images, and multi-exposure images, and was compared to existing state-of-the-art fusion methods by utilizing objective fusion quality metrics. The parameters of the GD method are further enhanced by employing the pattern search (PS) algorithm, resulting in an adaptive optimization strategy. Extensive experiments illustrated that the proposed GD fusion method ranked better on average than others in terms of objective quality metrics and CPU time consumption.

## 1. Introduction

The objective of image fusion is to merge the complementary information derived from multiple source images into a unified image [1,2,3,4]. In multi-modal medical image fusion, two or more images from different imaging modalities are combined [5]. Magnetic resonance (MR) and computed tomography (CT) are two different medical imaging modalities that have complementary strengths and weaknesses. CT images have high spatial resolution, which makes bones more visible, while MR images have high contrast resolution, which reveals soft tissues such as organs [6]. Visible and infrared image fusion is a computational technique that includes combined information from infrared and visible spectrum images to improve the visibility of objects and enhance the contrast of images, especially for enhanced night vision, remote sensing and pan-sharpening [7,8,9,10,11,12]. Multi-exposure image fusion involves the integration of multiple images, each captured at varying exposure levels, to generate a high-dynamic-range (HDR) image. HDR images retain details in both the dark and bright regions, which enhances image quality, increases visual fidelity, and improves image analysis in computer vision tasks [13,14]. Multi-focus image fusion is employed to merge multiple images exhibiting distinct focus levels into a singular composite image [15,16,17,18,19]. This results in improved overall sharpness, enhanced depth of field, and enhanced visual perception [20]. These benefits enable more accurate analysis and interpretation of the fused image in computer vision applications.

### 1.1. Related Work

Image fusion methods in the literature can be basically divided into two categories: pixel domain and transformation domain [21]. Pixel-domain (or spatial-domain) techniques combine the source images directly using their gray-level or color pixel values. The best-known example of this technique is the arithmetic averaging of source images. Arithmetic averaging can be used to combine both multi-sensor and multifocal images, but the biggest disadvantage of this method is that it reduces image contrast [22]. The basic idea of multi-scale, transform-based image fusion methods is applying a multi-resolution decomposition to each source image, combining the decomposition results with various rules to create a unified representation, and finally, applying an inverse multi-resolution transform [23]. Well-known examples of these approaches include principal component analysis (PCA), discrete wavelet transform (DWT), Laplacian pyramid (LP), and other pyramid-based transformations [24]. In recent years, several image fusion algorithms based on machine learning and deep learning approaches have been proposed [3,25,26,27,28]. These methods are robust and demonstrate superior performance. However, the training phase requires powerful, high-performance computing systems and plenty of input training data. Moreover, the trained models can be time-consuming for real-time applications [29].

Pixel level, feature level, and decision level are the three levels at which image fusion can take place. Pixel-level fusion directly integrates the original data from the source images to produce a fused image that is more informative for both computer processing and human visual perception. Compared to other fusion approaches, this approach strives to improve the visual quality and computing efficiency of the fused image. Li et al. proposed a pixel-based method by calculating the pixel visibility for each pixel in the source images [30]. Yang and Li proposed a multi-focus image fusion method based on spatial-frequency and morphologic operators [31]. Typically, in pixel-level image fusion, the weights are determined based on the activity level of various pixels [32]. In these studies, neural networks [33] and support vector machines [34] are employed to select pixels with the most significant activity, using wavelet coefficients as the input features. Ludusan and Lavialle proposed a variational pixel-based method for image fusion based on error estimation theory and partial differential equations to mitigate the noise of images [35]. In [36], a technique for multi-exposure image fusion is introduced which involves two primary stages: image features, including local contrast, brightness, and color dissimilarity, are computed to generate weight maps that are further improved using recursive filtering. Subsequently, the fused image is formed by combining the source images using a weighted sum based on these refined weight maps. Besides the many pixel-level methods available, region-based spatial methods that use blocks [37] or adaptive regions [38,39] have also been proposed to outperform existing methods.

Within the framework of anisotropic diffusion filter (ADF)-based image fusion algorithms, weight map layers are formed via image smoothing, which employs an edge protection method. These weight map layers undergo subsequent processing prior to the application of the fusion rule, culminating in the attainment of the final output [40]. Kumar has introduced the cross-binary filter (CBF) method, which takes into account both the gray-level similarity and geometric closeness of neighboring pixels without anti-aliasing. The source images are combined according to the weighted average, using the weights calculated from the detailed images extracted from the source images by the CBF method [41]. The fourth-order partial differential equations (FDPE) method first applies differential equations to each source image to obtain approximate images. Then, PCA is used to obtain optimum weights for the detailed images, which are then combined to obtain the final, detailed image. The ultimate approximation of the image is derived by performing an averaging operation on the set of approximate images. Subsequently, the fused image is computed by merging the final approximation with the detailed images [42]. The context enhancement (GFCE)-based method preserves the details in the visible input image and the background scene. Thus, it can successfully transfer important IR information to the composite image [43]. The gradient transfer fusion (GTF) method, which is based on gradient transmission and total variation (TV) minimization, tries to maintain appearance information and thermal radiation simultaneously [44]. The hybrid multi-scale decomposition method (HMSD) decomposes the source image into very distant texture details and edge features using a combination of bilateral filters and the versatile Gaussian method. This offset allows us to better capture important very sensitive IR spectral features and separate fine texture details from large edges [45]. The infrared feature extraction and visual information preservation (IFEVIP) method provides a simple, fast, but effective fusion of infrared and visual images. Firstly, the reconstruction of the infrared background is accomplished by leveraging quadtree decomposition and Bézier interpolation. Subsequently, the extraction of bright infrared features is performed by subtracting the reconstructed background from the infrared image, followed by a refinement process that reduces redundant background information [46]. The multi-resolution singular value decomposition (MSVD) method is an image fusion technique based on a process that bears a resemblance to wavelet transform and involves filtering the signal independently using low-pass and high-pass finite impulse response (FIR) filters, followed by the decimation of the output of each filter by a factor of two to achieve the first level of decomposition [47]. The VSMWLS approach, designed to enhance the transfer of significant visual details while minimizing the inclusion of irrelevant infrared (IR) details or noise in the merged image, represents a multi-scale fusion technique that incorporates visual salience maps (VSM) and weighted least square (WLS) optimization [48]. Liu et al. proposed an approach based on deep convolutional neural networks (CNN) for both infrared–visible image fusion [49] and multi-focus image fusion [50]. They successfully addressed the crucial issues of activity level measurement and weight assignment in image fusion by using a Siamese convolutional network to construct a weight map by integrating pixel activity information from two source images [49]. On the other hand, because focus estimation and image fusion are two distinct problems, traditional image fusion techniques sometimes struggle to perform satisfactorily. Liu et al. suggest a deep learning method that avoids the requirement for separate focus estimation by learning a direct mapping between source images and a focus map [50].

### 1.2. Contributions of This Study and Advantages of the Proposed Method

To overcome the limitations of the existing image fusion methods, a simple and efficient general image fusion technique named Gaussian of differences (GD) is proposed. The unique aspects of the proposed GD image fusion method can be listed as follows:

- The proposed algorithm does not use any transformations and works directly in the pixel domain. Also, it is based on basic image convolution and linear weighting, which makes it simple and efficient. It can be implemented on real-time systems and is suitable for parallel processing.
- The method enhances the high-frequency components of each input image using simple first-order derivative edge detection. It then uses a Gaussian filter to weight the contributions of neighboring pixels to the center pixel, with the weight decreasing with distance.
- The proposed GD method has only two control parameters: the size of the filter and the standard deviation of the distribution. In addition to making use of predefined parameters, an optimal solution using the pattern search (PS) algorithm is also proposed to investigate the adaptability capability of the GD method.
- The method is a general-purpose image fusion algorithm that can be used in a variety of applications, including multi-modal medical image fusion, infrared and visible image fusion for enhanced night vision or remote sensing, multi-focus image fusion for extending the depth of field, and multi-exposure image fusion for high dynamic range imaging.
- It can combine single-band (gray-level), color (RGB), multi-spectral, and hyperspectral images due to its generalized structure.

The rest of this paper is organized as follows: the proposed GD fusion method is briefly introduced, illustrated, and demonstrated in Section 2. Section 3 outlines extensive experiments with 48 pairs of test images (in total) belonging to four different image fusion applications. Finally, Section 4 concludes the paper.

## 2. Proposed Method

Speed and performance are crucial features of imaging systems. Therefore, one of the primary factors considered in designing the proposed image fusion method was keeping the computational complexity low. Another significant concern was the generation of a single composite image that incorporates meaningful information from images captured at multiple or diverse wavelengths [51]. The resulting combined image should be suitable for both human interaction and computer vision applications [52].

Many of the existing fusion methods in the literature employ multi-resolution transforms such as DWT, LP, and discrete cosine transform (DCT) to mitigate the impact of image misalignments [53]. However, these transformations increase the computational complexity of the methods. Edge information, which typically contains high-frequency components, plays a crucial role in determining the importance of pixels in an image.

**Figure 1:**
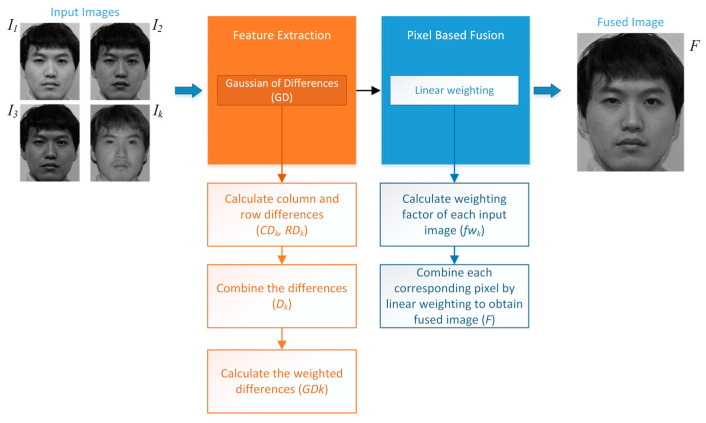
Proposed general image fusion method based on pixel-based linear weighting using the Gaussian of differences (GD).

For the method proposed in this paper, at first, the gradients of each source image based on the first-degree derivative information are computed. These gradients are then evaluated along with the neighboring pixels. Linearly, the contribution of each pixel from different input images to the resulting pixel in the final fused image is determined. The block diagram of the proposed GD image fusion method is presented in Figure 1.

**Figure 2:**
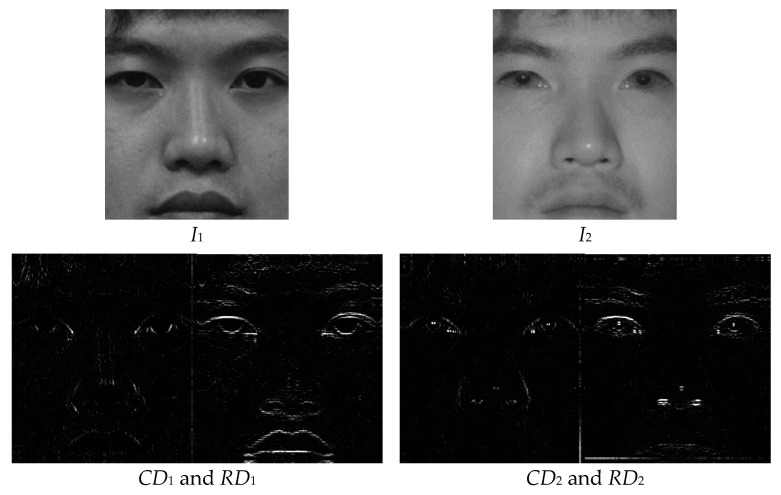
Sample input images (*I*_1_ and *I*_2_) [54] and their column and row differences.

The steps of the proposed GD fusion method can be summarized as follows:

1. Edge information is generally related with the information content of an image. The first-order derivation (difference of adjacent pixels) of an image simply emphasizes the edges. The column and row differences of each input image are calculated:
  (1) $$CD_{k}\left(i,j\right)={\left[I_{k}\left(i,j\right)-I_{k}\left(i,j+1\right)\right]}^{2}\;RD_{k}\left(i,j\right)={\left[I_{k}\left(i,j\right)-I_{k}\left(i+1,j\right)\right]}^{2}$$

where *i* and *j* are row and column indexes, *CD* and *RD* indicate the column and row differences, respectively, and k is the input image index. In Figure 2, a face image in the visible spectrum is given as *I*_1_ and an infrared image of the same scene is given as *I*_2_. The column and row differences of the input images are also visualized.

**Figure 3:**
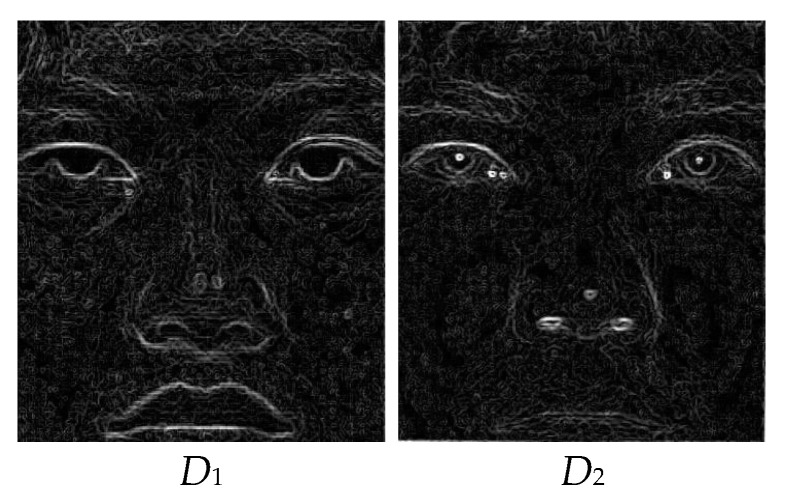
Combined difference images (*D*) of the input images.

**Figure 4:**
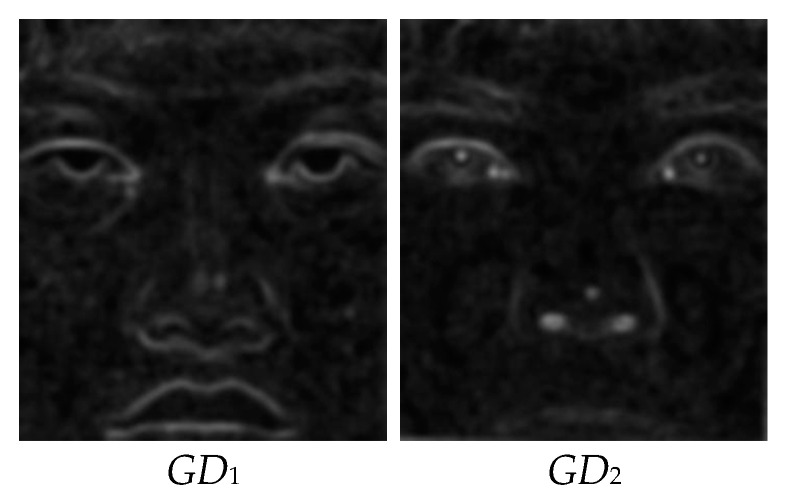
Gaussian of differences (*GD*) of the input images.

2. Column and row differences emphasize the edges along vertical and horizontal axes, respectively. To combine them into a single representation (*D*), the Euclidian distance is used, and features related with each pixel based on the edge content are calculated (visualized in Figure 3):
  (2) $$D_{k}\left(i,j\right)=\sqrt{CD_{k}\left(i,j\right)+RD_{k}\left(i,j\right)}$$

3. Linear weighting is a well-known approach used to determine the information transfer of each input image to the output fused image. To determine the contributions of neighbors of pixels in each image at different input images to the information content of the respective pixel, the differences are filtered (i.e., weighted) using a 2D Gaussian filter and the Gaussian of the Differences is obtained (*GD*), which is visualized in Figure 4. This representation will be used to calculate the weighting factor of each pixel:
  (3) $$GD_{k}\left(i,j\right)=\overset{s}{\underset{p=-s}{\sum }}\overset{s}{\underset{r=-s}{\sum }}w\left(p,r\right)\cdot D_{k}\left(i+p,j+r\right)$$

where *s* is the window size, w is a 2D Gaussian filter with a standard deviation of σ:

(4) $$w=\frac{1}{2\pi \sigma^{2}}e^{-\left(x^{2}+y^{2}\right)/2\sigma^{2}}$$

**Figure 5:**
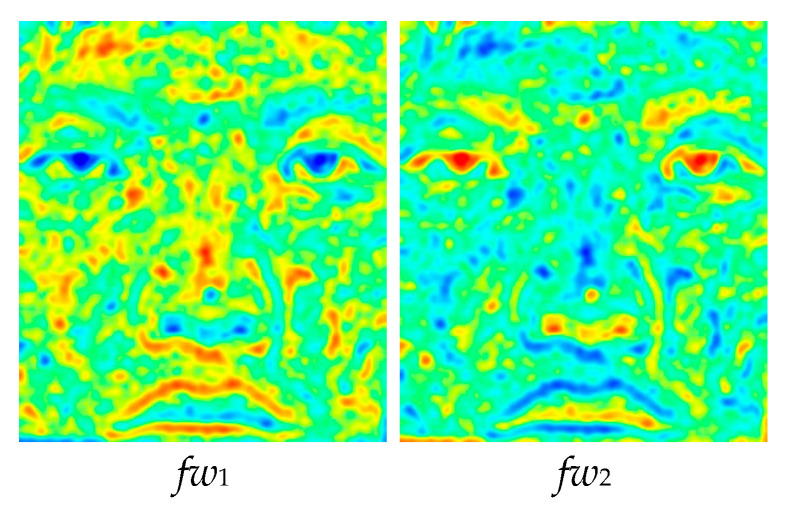
Weighting factors (*fw*) for the input images.

**Figure 6:**
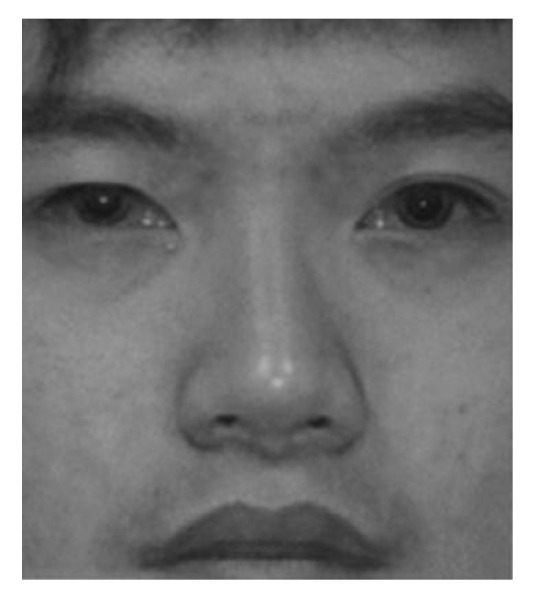
Fused image (*F*).

4. Weighting factors (*fw*) are determined for the pixels in each input image using *GD* proportional to their values, as visualized in Figure 5. Therefore, the sum of the weighting coefficients of a specific pixel is always equal to one, regardless of how many input images exist:
  (5) $$fw_{k}\left(i,j\right)=\frac{GD_{k}\left(i,j\right)}{\sum GD_{k}\left(i,j\right)}$$

5. The fused image (*F*), as demonstrated in Figure 6, is created with the linear weighting method using weighting factors. Assume that there are two input images in an application, and for a specific pixel, let the *fw*s be 0.4 and 0.6, respectively. The fusion result of that specific pixel is summation of 40% of the first input image’s pixel value $I_{1}(i,j$) and 60% of the second input image $I_{2}(i,j$).
  (6) $$F\left(i,j\right)=\sum fw_{k}\left(i,j\right)\cdot I_{k}\left(i,j\right)$$

**Figure 7:**
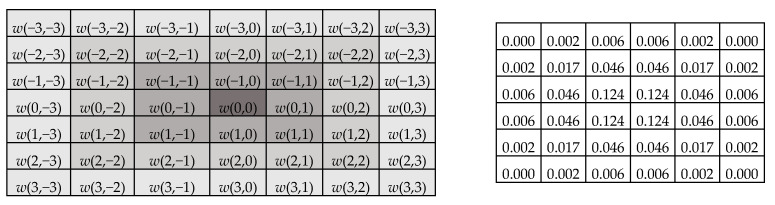
Gaussian kernel (*w*) for *s* = 3 and σ=1.

In the prosed GD fusion method, before calculating the contribution of pixels to the fused image, the placement of the Gaussian filter (7 × 7 for *s* = 3) is used to contribute to the edge information of each pixel. This is given in Figure 7. The pixel of interest in the center is weighted with the highest coefficient *w*(0,0) in the Gaussian kernel, and the neighbors are weighted with smaller coefficients as they move away from the center due to the nature of the Gaussian kernel.

The fusion results are promising, as shown in the visual steps of the proposed GD method. In Step 1, the column and row differences are calculated, and the edge content, which exhibits the high-frequency components of the input images, is obtained, as shown in Figure 2. In Step 2, the row and column differences are combined with the help of the Euclidean distance, and the results for the sample images are given in Figure 3. In the third step of the method, the edge information, obtained using the differences of each pixel, is convolved with the Gaussian kernel with *s* = 10 in order to include the contribution of the neighbors of the relevant pixel. The GDs obtained are shown in Figure 4. In Step 4, weighting factors are obtained using GDs and visualized in Figure 5 using the jet coloring map. Here, the red color indicates that the numerical value of the weighting factor for the relevant pixel is one, which is the highest ratio, and the blue color indicates that the lowest value is zero. When the weighting factor matrix (*fw*_1_) of the visible image is examined, the outer edges of the lips, nose, and eyes are enhanced. On the other hand, when the weighting factor matrix (*fw*_2_) of the near-infrared image is examined, details such as the iris and nostrils seem to have higher factors. The fused image (*F*), obtained in the fifth step of the method with the weighted average using the weighting factors, is given in Figure 6. When the final fused image is examined, it can be seen that the details that are present in the visible image but not in the infrared image, and vice versa, are combined into a single composite image.

### Optimization of GD Parameters

A Gaussian filter is defined by two parameters, as given in Equation (4): the size of the filter (*s*) and the standard deviation of the Gaussian distribution σ. Using predefined values for *s* and σ may not be suitable for all images. Therefore, an optimal approach to determine the best parameter set for any input image is proposed in this section.

**Figure 8:**
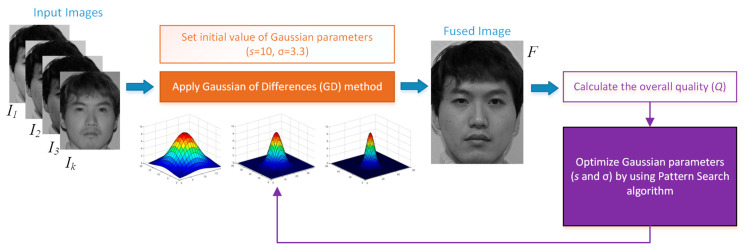
Optimization of the parameters of the proposed GD fusion method.

A block diagram of the proposed optimal scheme is illustrated in Figure 8. As can be seen in the figure, pattern search (PS) is chosen as the optimizer due to its simplicity and robustness. Also, PS is a well-known, derivative-free algorithm that does not require a gradient [55]. The steps of the proposed Gaussian of differences with pattern search (GDPS) method can be summarized as follows:

1. Define the maximum iteration number of PS and set the initial values of GD parameters.
2. Evaluate the initial solution and calculate its fitness value (overall quality of the fused image):
  a. Apply all steps of the proposed GD fusion method explained in the previous section (Equations (1)–(6)).
  b. Calculate the fused image quality using an image metric (see Section 3.3).

(7) $$fitness=Q\left(F\left(s,\sigma \right)\right)$$

where *Q* is the image quality metric to be maximized, *F* is the fused image, *s* is the size of the Gaussian filter, and σ is the standard deviation of the Gaussian distribution.

3. Apply the operators of PS to find a better GD parameter solution that maximizes the fused image quality.
4. Repeat Steps 2 and 3 until the maximum iteration number or a predefined stopping condition is reached.

## 3. Experimental Results

For this section, a comprehensive series of experiments were conducted to assess the performance of the proposed GD method. As explained in Section 2, the GD method has only two control parameters: the size of the Gaussian kernel (*s*) and the standard deviation of the Gaussian distribution (σ). In the experiments, two types of cases were evaluated:

- First, a predefined parameter set for GD was used. *s* values of 5, 10, and 15 values, named GD5, GD10, and GD15, respectively, were evaluated. In this case, the second parameter σ was defined according to the value of the filter size, σ=s/3.
- Second, the parameters of GD were adaptively determined by using the pattern search optimization algorithm to maximize the image quality. Unreported intensive experiments have shown that using Qabf, Q_cb_, and Q_cv_ as fitness functions generates the best results. Therefore, the versions of this case were named GDPSQABF, GDPSQCB, and GDPSQCV, respectively.

### 3.1. Image Dataset

To validate the performance of the proposed GD method, four different types of image fusion cases were selected: multi-modal medical images [56], multi-sensor infrared and visible images [45], multi-focus images [57], and multi-exposure images [58]. The specifications of the images used the experiments are summarized in Table 1.

**Figure 9:**
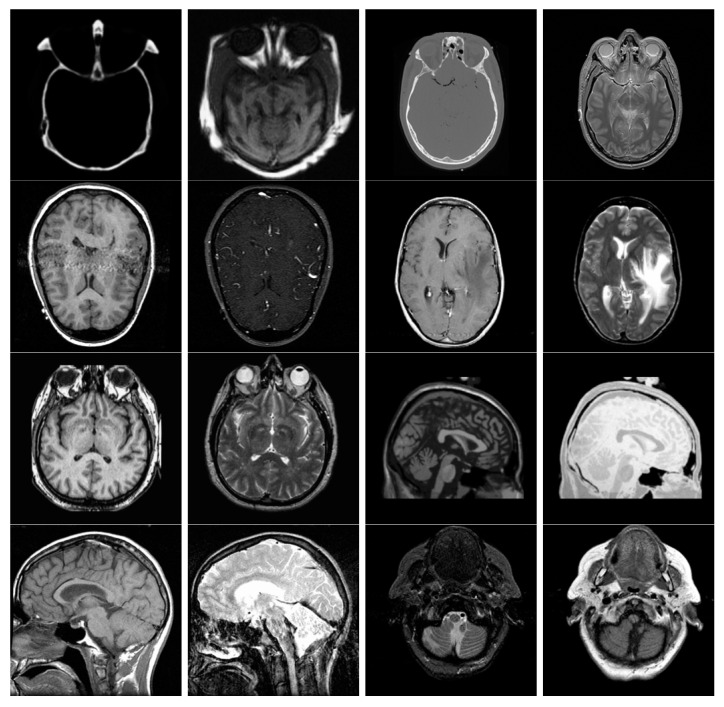
Multi-modal medical images used in the experiments.

**Figure 10:**
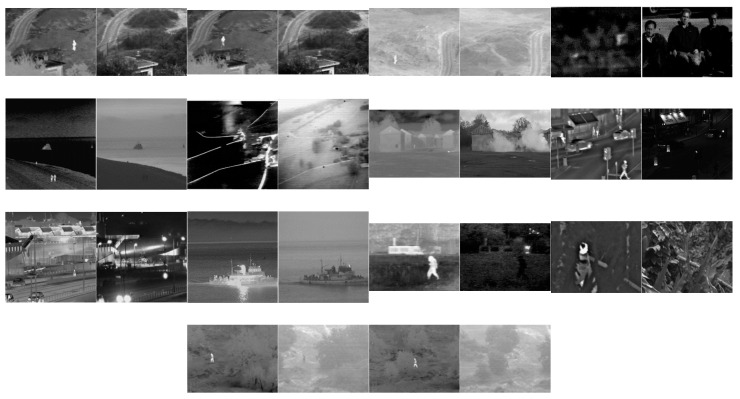
Multi-sensor infrared and visible images used in the experiments.

**Figure 11:**
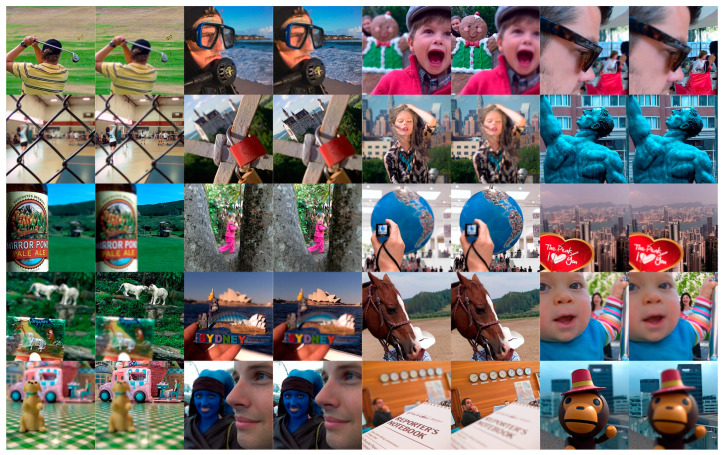
Multi-focus images used in the experiments.

**Figure 12:**
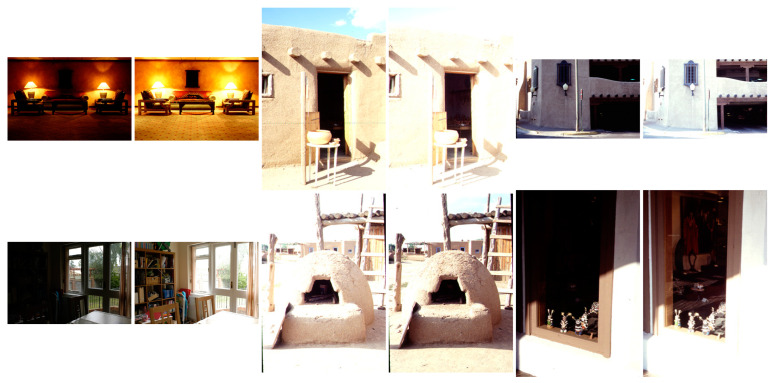
Multi-exposure images used in the experiments.

The multi-modal medical image dataset had eight pairs of images, which are shown in Figure 9. The multi-sensor infrared and visible image dataset had 14 pairs of images, which are shown in Figure 10. The multi-focus dataset had 20 pairs of images, which are shown in Figure 11. And the multi-exposure image dataset had six pairs of images, which are shown in Figure 12.

### 3.2. Experimental Setup

The environmental features of the experiments are summarized in Table 2. Since there is no training phase in the proposed method, a standard workstation could be sufficient. In the experiments, the MATLAB library developed by Zhang et al., published openly on GitHub, was used [59].

The configuration parameters of the fusion methods used in the experiments for comparison are summarized in Table 3. For the comparison methods, the default parameters of the original authors were used. For the proposed GD method, the parameters were determined by trial and error. Therefore, six different cases of the proposed GD method were included in the experiments (GD5, GD10, GD15, GDPSQABF, GDPSDQCB, and GDPSQCV) to emphasize the stability and adaptability of our method.

The experiments were conducted on 48 pairs of images. However, due to lack of space, only eight image pairs were selected to be visualized and compared in detail in the following sections. To investigate all results, please see the Supplementary Materials section at the end of the paper.

### 3.3. Objective Quality Metrics

Except for the visual analysis of the fusion results, objective quality metrics were utilized to compare the proposed method with other methods quantitatively [60]. The evaluation of a fused image by visual inspection included steps such as assessing the clarity and sharpness of the output image and identifying the amount of information transferred from input images to the source image. Visual evaluation is a very helpful method for comparing performances; however, visual interpretation is highly subjective. In order to make a fair comparison, the following image quality criterions were used in the experiments:

Entropy (EN) is a metric that is used the measure the information content of an image [61]:

(8) $$\mathrm{EN}\left(I_{f}\right)=\sum_{x=0}^{L}h_{I_{f}}\left(\chi )\log h_{I_{f}}(\chi \right)$$

where *L* is the number of gray levels and $h_{I_{f}}$*(i)* is the normalized histogram of the fused image.

Mutual information (MI) is a numerical metric that measures the interdependence of two variables. It is used to measure the amount of information shared by two images. The MI for two discrete random variables *U* and *V* is defined by [62]:

(9) $$\mathrm{MI}(U,V)=\sum_{v\epsilon V}\sum_{u\epsilon U}p\left(u,v\right)\log\frac{p\left(u,v\right)}{p\left(u\right)p\left(v\right)}$$

where $p\left(u,v\right)$ indicates the probability density function of *U* and *V*, and $p\left(u\right)$ and $p\left(v\right)$ are the marginal probability density functions of *U* and *V*, respectively.

The peak signal-to-noise ratio (PSNR) represents the logarithmic decibel scale ratio between the maximum potential power of a signal and the power of the noise that introduces distortion to said signal. A high PSNR value indicates high image quality. *L* is the number of colors in the gray level and is taken as 255 [63]:

(10) $$\mathrm{PSNR}\;(f,g)=10\log_{10}\left(\frac{L^{2}}{\frac{1}{M\times N}\sum_{f=1}^{M}\sum_{g=1}^{N}{\left(R\left(f,g\right)-F\left(f,g\right)\right)}^{2}}\right)$$

Edge-based similarity (Qabf) is obtained by weighting the normalized edge information of both source images [64]:

(11) $$\mathrm{Qabf}=\frac{\sum_{n=1}^{N}\sum_{m=1}^{M}Q^{AF}\left(n,m\right)w^{A}\left(n,m\right)+Q^{BF}\left(n,m\right)w^{B}\left(n,m\right)}{\sum_{i=1}^{N}\sum_{j=1}^{M}\left(\;w^{A}\left(i,j\right)+w^{B}\left(i,j\right)\right)}$$

The structure similarity index method (SSIM) is a metric with the purpose of measuring how much of the structure of the input image is preserved in the fused image [65]:

(12) $$\mathrm{SSIM}(x,y)=\frac{\left(2\mu_{x}\mu_{y}+c_{1}\right)\left(2\sigma_{xy}+c_{2}\right)}{\left(\mu_{x}^{2}+\mu_{y}^{2}+c_{1}\right)\left(\sigma_{x}^{2}+\sigma_{y}^{2}+c_{2}\right)}$$

The Chen–Blum metric (Q_cb_) is a referenceless image quality metric inspired by human perception [66]. The Q_cb_ value is obtained by calculating the average value of the global quality map:

(13) $$Q_{\mathrm{cb}}(x,y)=\lambda_{A}(x,y)\;Q_{AF}(x,y)+\lambda_{B}\left(x,y)\;Q_{BF}(x,y\right)$$

Cross entropy (CE) serves as a metric to assess the congruity of the information content between the input images and the fused image. Reference and fused images including the same information will have a low CE value [67]:

(14) $$\mathrm{CE}(I_{1},\;I_{2}:I_{f})=\frac{CE\left(I_{1},I_{f}\right)+CE\left(I_{2},I_{f}\right)}{2}$$

Root mean square error (RMSE) is a measure of accuracy used to realize differences in estimation errors from different estimators for a variable and is desired to be as low as possible [63]:

(15) $$\mathrm{RMSE}=\sqrt{\frac{1}{MN}\sum_{i=1}^{M}\sum_{j=1}^{N}{\left(I_{a}\left(i,j\right)-I_{b}\left(i,j\right)\right)}^{2}}$$

Chen Varshney (Q_cv_) is a quality metric used in image fusion based on regional information inspired by human perception [68]. The lower the Q_cv_, the better the fusion result:

(16) $$Q_{\mathrm{cv}}=\frac{\sum_{I=1}^{N}\sum_{I=1}^{L}\left(\lambda \left(X_{I}^{W_{1}}\right)D\left(X_{I}^{W_{1}},X_{F}^{W_{1}}\right)\right)}{\sum_{I=1}^{N}\sum_{I=1}^{L}(\lambda \left(X_{I}^{W_{1}}\right))}$$

where *X* = [$X_{1}$, $X_{2}$ …, $X_{N}$] input images and $X_{F}$ is the fused image.

For the EN, MI, PSNR, Qabf, SSIM, and Q_cb_ metrics, higher values indicate better results. And for CE, RMSE, and Q_cv_, lower values indicate good performance. In the following tables, the best result is colored in green, second-best result is colored in dark red, and the third-best result is indicated by a blue color.

### 3.4. Medical Image Fusion

**Figure 13:**
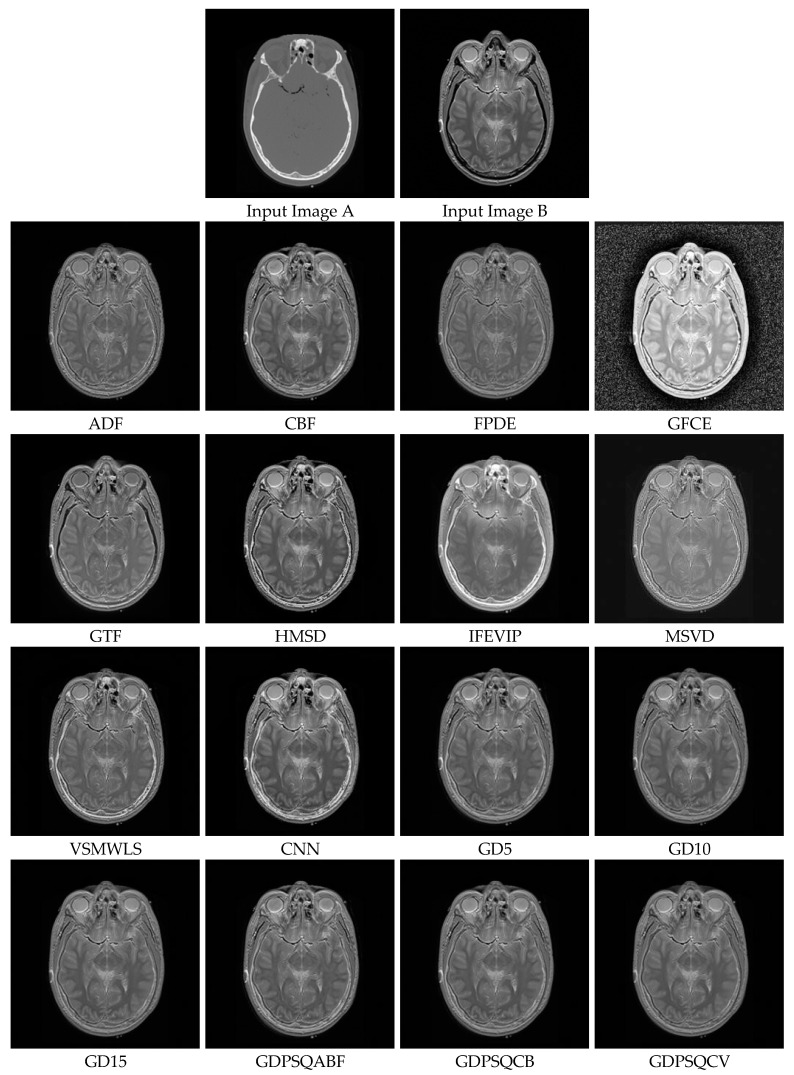
Medical image set M#2 (Images A and B) and their fusion image results, obtained using comparison methods.

For this sub-section, medical images M#2 and M#5, shown in Figure 9, were selected from eight candidates among the dataset and tested. The visual fusion results of image set M#2 are given in Figure 13. Input Image A is a computed tomography (CT) slice image of the human brain, and Image B is a magnetic resonance (MR) image of the same section. In an ideal case, the bright bone features shown in the CT image and the tissue features shown in the MR image should be included in the fused image. As can be seen from the visual results, the GFCE image has obvious noise in the background. The FPDE and MSVD images lack contrast. The IFEVIP and VSMWSL images resemble mostly Input A (CT) and ignore Input B (MR). As a result, the ADF, CBF, GTF, HMSD, and proposed GD methods show better visual performance than others.

In Table 4, the numerical results of the quality metrics of the comparison methods for M#2 are given. As can be seen in the table, the VSMWLS, proposed GD15, and proposed GDPSQCV methods show better performance according to the numerical metrics. On the other hand, GFGC, ADF, and IFEVIP show the worst performance compared to the others.

**Figure 14:**
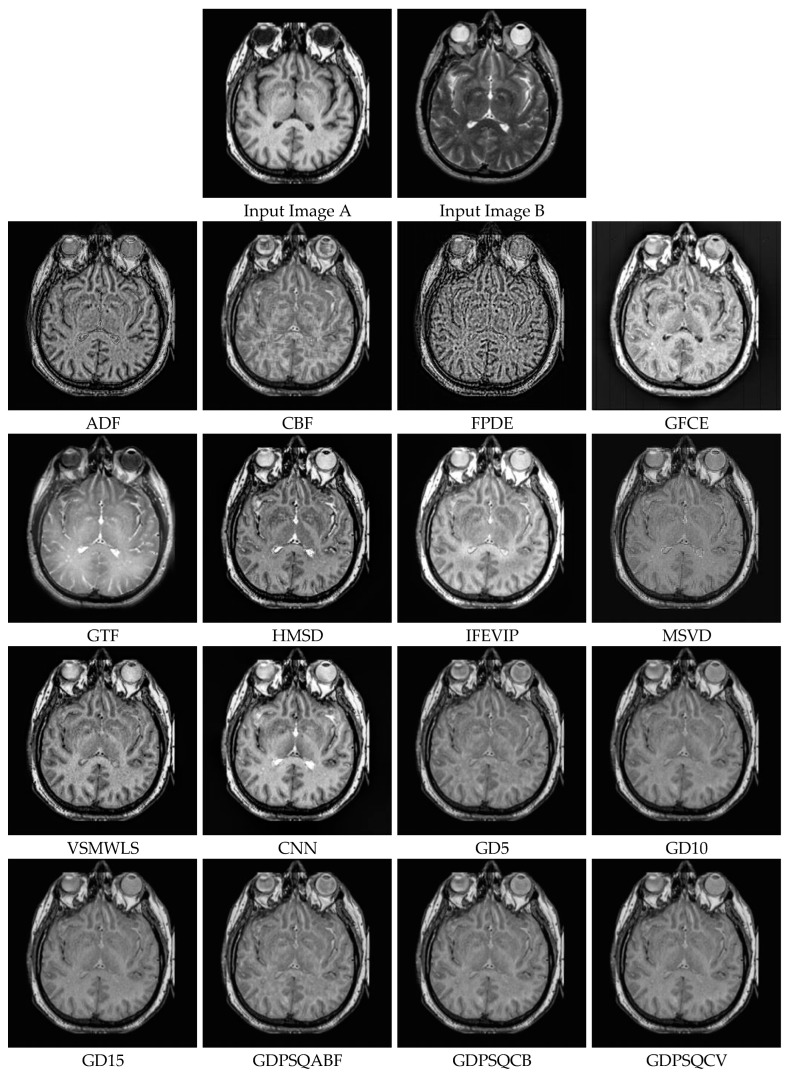
Medical image set M#5 (Images A and B) and their fusion image results, obtained using comparison methods.

The results of the image set M#5 are given in Figure 14. As can be seen from the results, ADF, FPDE, GFCE and MSVD show poor visual performance. On the other hand, the CBF, VSMWLS, and proposed GD methods show better visual performance than other techniques.

In Table 5, the numerical results of the quality metrics of the comparison methods for M#5 are given. As can be seen in Table 5, the CNN, proposed GD10, and proposed GDPSQCV methods show better performance according to the numerical results. On the other hand, MSVD, FPDE, and GFCE show the worst performance compared to the others.

### 3.5. Infrared and Visible Image Fusion

**Figure 15:**
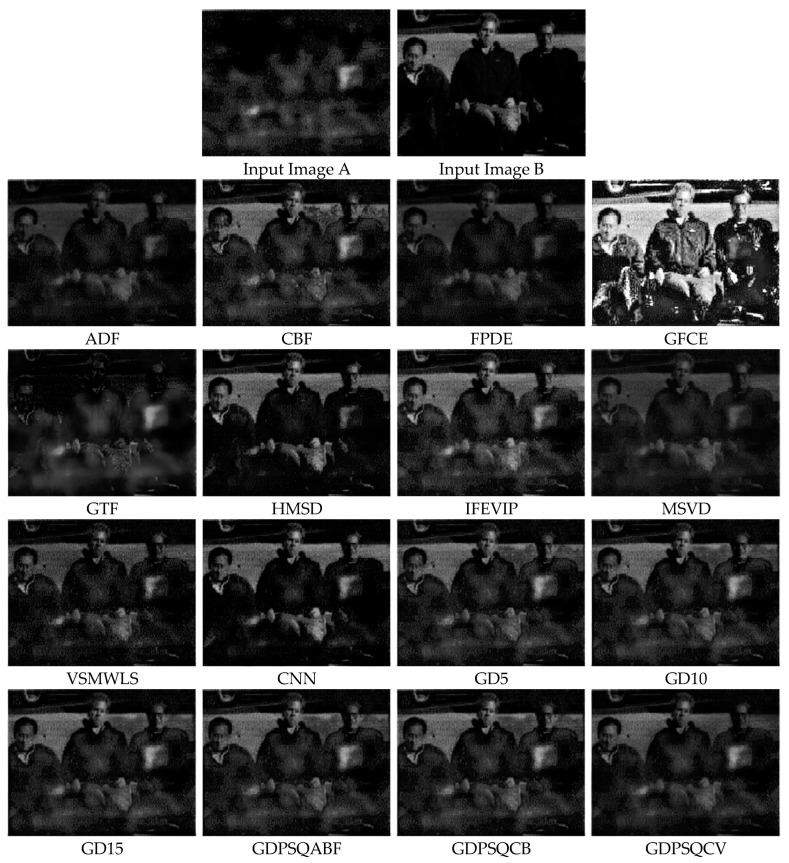
Infrared and visible image set IV#4 (Images A and B) and their fusion image results, obtained using comparison methods.

Infrared images acquired at wavelengths of 750 nm–1 mm reveal the thermal radiation of objects in a scene. On the other hand, RGB color images are captured at 400 nm–750 nm wavelengths, a range which is called the visible spectrum. For this sub-section, infrared and visible images IV#4 and IV#5, shown in Figure 10, from 14 candidates among the dataset were selected and tested. The visual fusion results of image set IV#4 are given in Figure 15. Input Image A is an infrared image of a scene that depicts three people, with a gun being held by the person on the right. Image B is a visible image of the same scene. Ideally, both thermal and visible features should be included in the fused image. As can be seen from the visual results, the contrast of the GFCE image is saturated. The result of the GTF method is blurry and includes very few features from the visible image input. The result of the MSVD method has low contrast. On the other hand, the CBF, ADF, VSMWSL, CNN, and proposed GDPS methods show better performance than the others.

From Table 6, it can be seen that CBF, VSMWSL, and the proposed GD15 and GDPSQCB methods show better performance according to the objective metrics. On the other hand, GFCE, GTF, and MSVD show the worst performance compared to the other methods.

**Figure 16:**
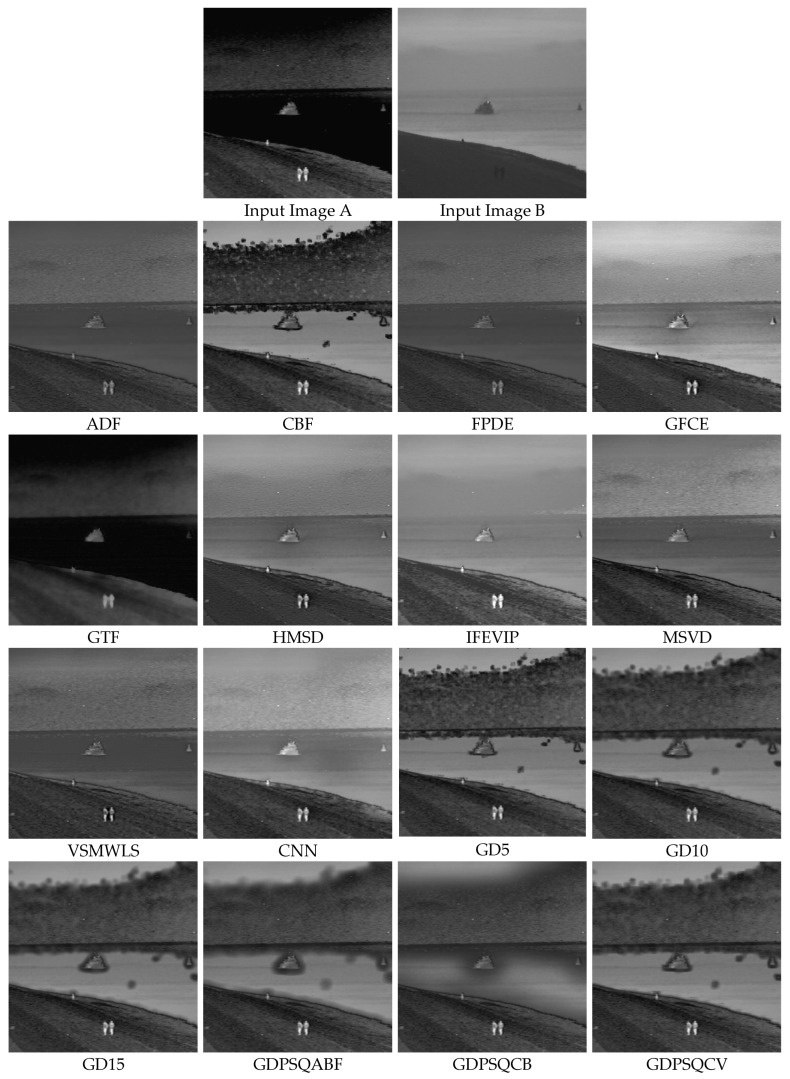
Infrared and visible image set IV#5 (Images A and B) and their fusion image results, obtained using comparison methods.

The results of image set IV#5 are given in Figure 16. As can be seen from the results, CBF, GTF, and all of the GD methods except GDPSQCB show poor visual performance. On the other hand, the HMSD and MSVD methods show better visual performance than the other techniques.

In Table 7, the quantitative fusion results are given. As can be seen, HMSD, MSVD, FPDE, and GDPSQABF show better performance according to the objective metrics. On the other hand, GFCE, GTF, and the proposed GD5, GD10, GD15, and GDPSQCV methods show the worst performance compared to the other methods.

### 3.6. Multi-Focus Image Fusion

Images captured using a single lens of scenes containing objects at different distances have blurry regions. To extend the depth of field, images with different focal lengths are fused.

**Figure 17:**
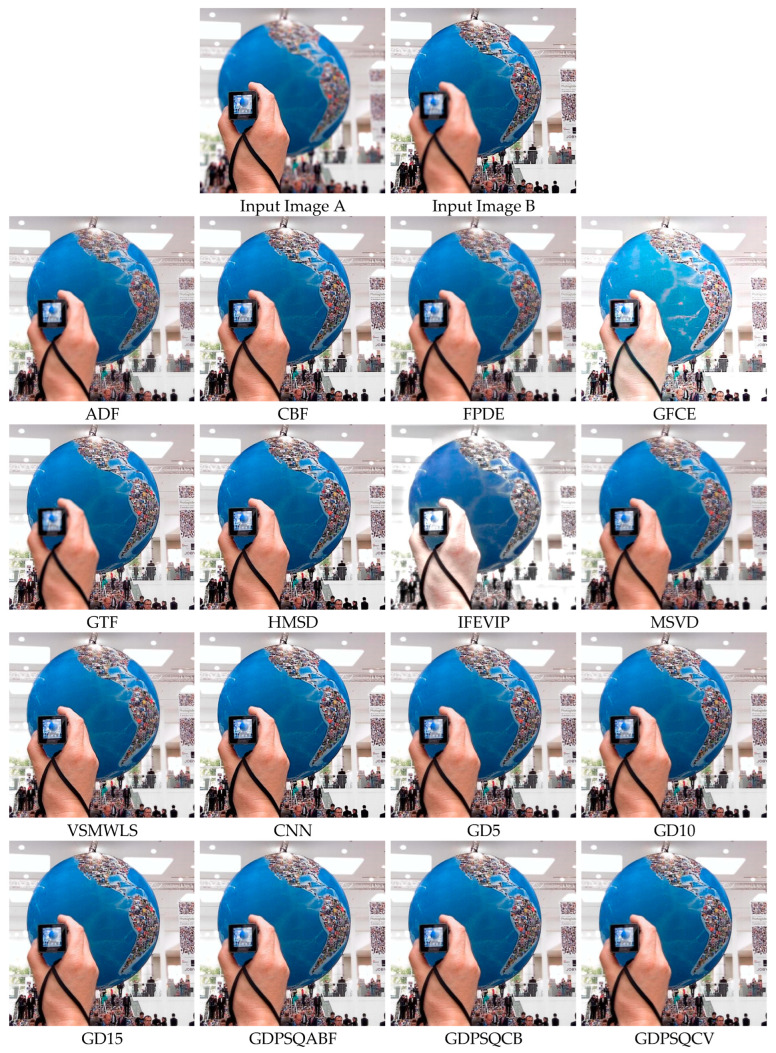
Multi-focus image set F#11 (Images A and B) and their fusion image results, obtained using comparison methods.

For this sub-section, multi-focus images F#11 and F#15, shown in Figure 11, from 20 candidates among the dataset were selected and tested. In Figure 17, the fusion results of test image F#11 are given. In Input Image A, the near objects (hand and camera) are in focus, while in Input Image B, the far object (globe) is in focus. An everywhere-in-focus image is desired, which the fused image provides.

The visual results show that the contrasts of the GFCE and IFEVIP images are saturated. The GTF result is blurry (hand and camera). The MSVD, ADF, and FPDE results are also not sharp (globe). On the other hand, CBF, HMSD, VSMWSL, CNN, and the proposed GDPS methods show better performance than the others.

In Table 8, the numerical results of the quality metrics of the comparison methods for F#11 are given. As can be seen in the table, CBF, CNN, and the proposed GD15, GD10, GDPSQCV, and GDPSQCB methods show better performance according to the numerical results. On the other hand, GFCE, IFEVIP, and MSVD show the worst performance compared to the others.

**Figure 18:**
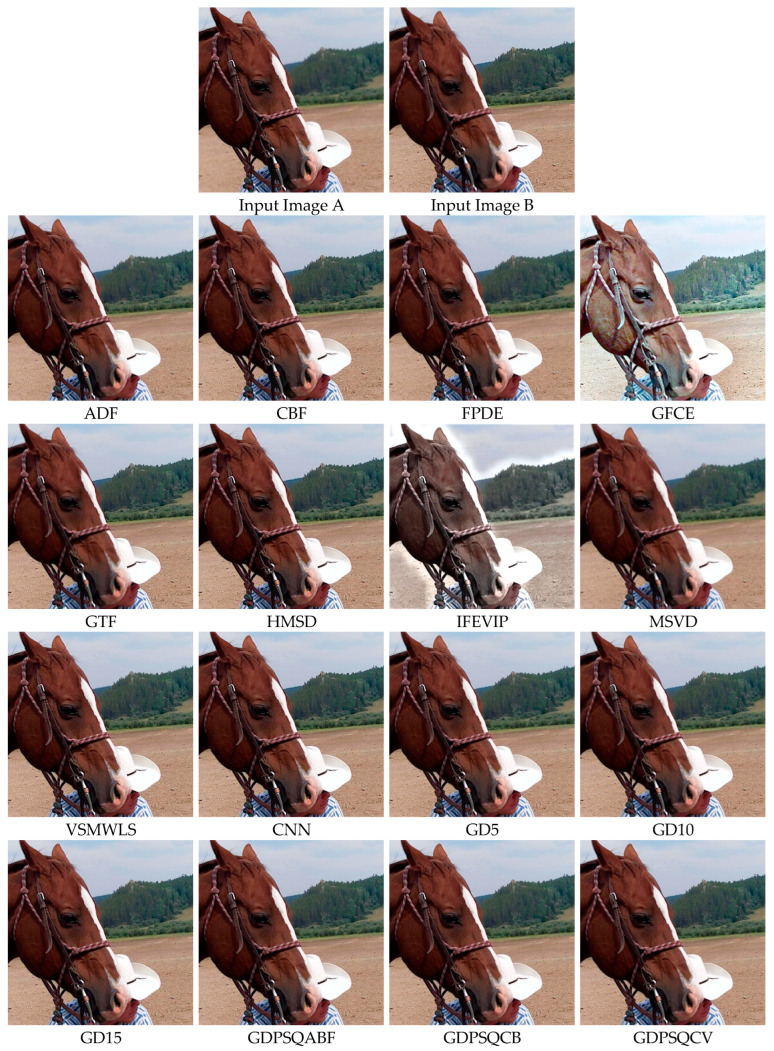
Multi-focus image set F#15 (Images A and B) and their fusion image results, obtained using comparison methods.

The results of image set F#15 are given in Figure 18. As can be seen from the results, IFEVIP and GFCE show very poor visual performance. The results of MSVD and GTF contain blurry regions. On the other hand, CBF, VSMWLS, HMSD, ADF, CNN, and the proposed GDPSQCB methods show better visual performance than the other techniques.

From Table 9, it can be seen that GTF, CBF, CNN, and the proposed GDPSQCB, GD15, and GD10 methods show better performance according to the objective metrics. On the other hand, GFCE, IFEVIP, and MSVD show the worst performance compared to other methods.

### 3.7. Multi-Exposure Image Fusion

**Figure 19:**
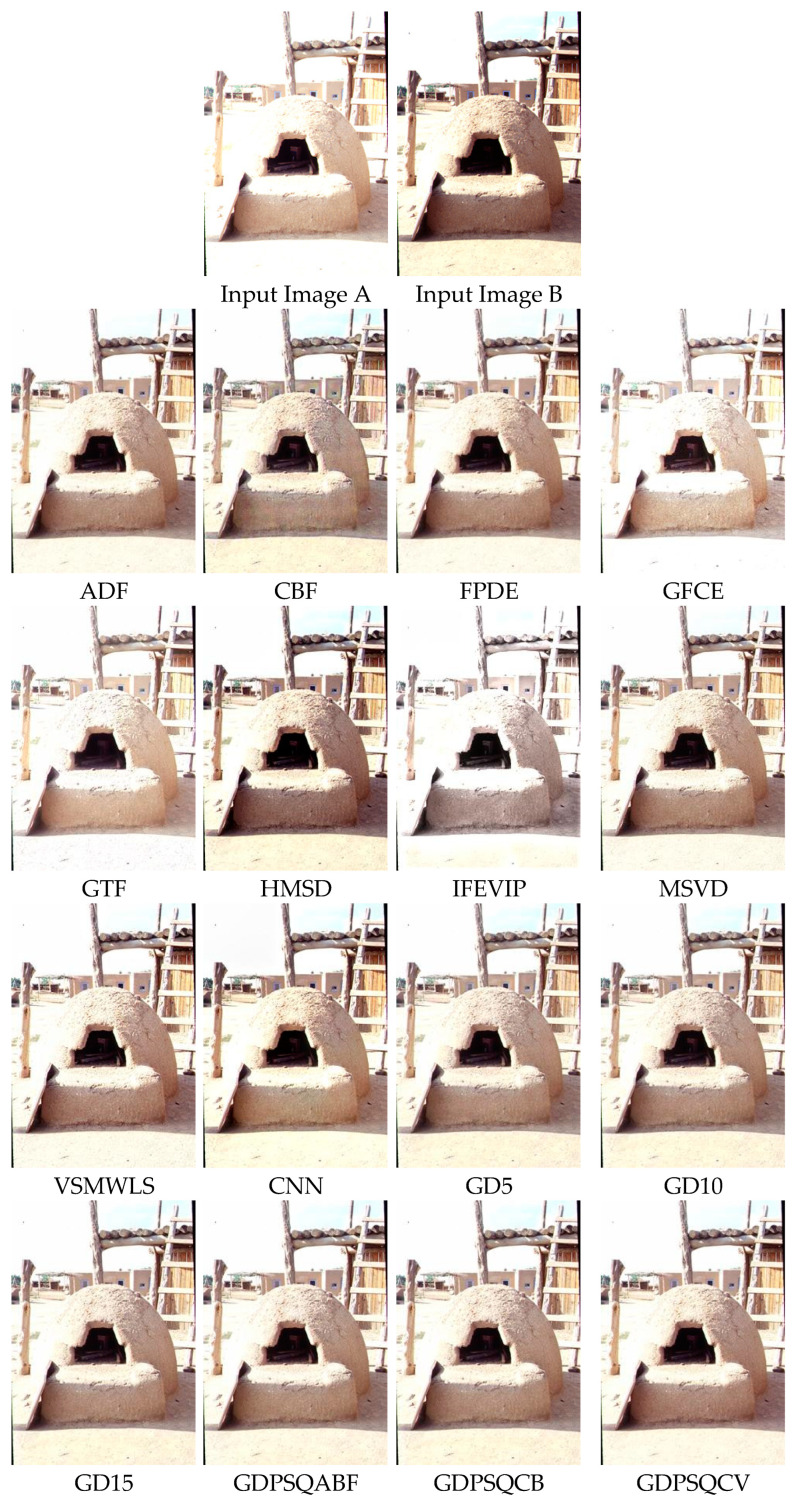
Multi-exposure image set E#5 (Images A and B) and their fusion image results, obtained using comparison methods.

In the last case, image fusion algorithms were compared with regard to their use on multi-exposure images selected from six candidates among the dataset (images E#5 and E#6 of Figure 12). For a first example, the visual results of image E#5 are given in Figure 19. In Input Image A, the inside of the oven is visible, and the remaining objects are saturated. However, in Input Image B, the background details are in good contrast. Multi-exposure image fusion helps us create a high-dynamic-range image in which whole regions have balanced contrast. As can be seen from the results, CBF, HMSD, VSMWLS, CNN, and the proposed GD methods exhibit good visual performance. Moreover, the IFEVIP, GFCE, and GTF methods show poorer visual performance than the other techniques.

In Table 10, the numerical results of the quality metrics of the comparison methods are given for image set E#5. As can be seen in the table, ADF, FPDE, and the proposed GD15 and GDPSQCV methods show better performance according to the numerical results. On the other hand, GFCE, IFEVIP, and GTF show the worst performance compared to the others.

**Figure 20:**
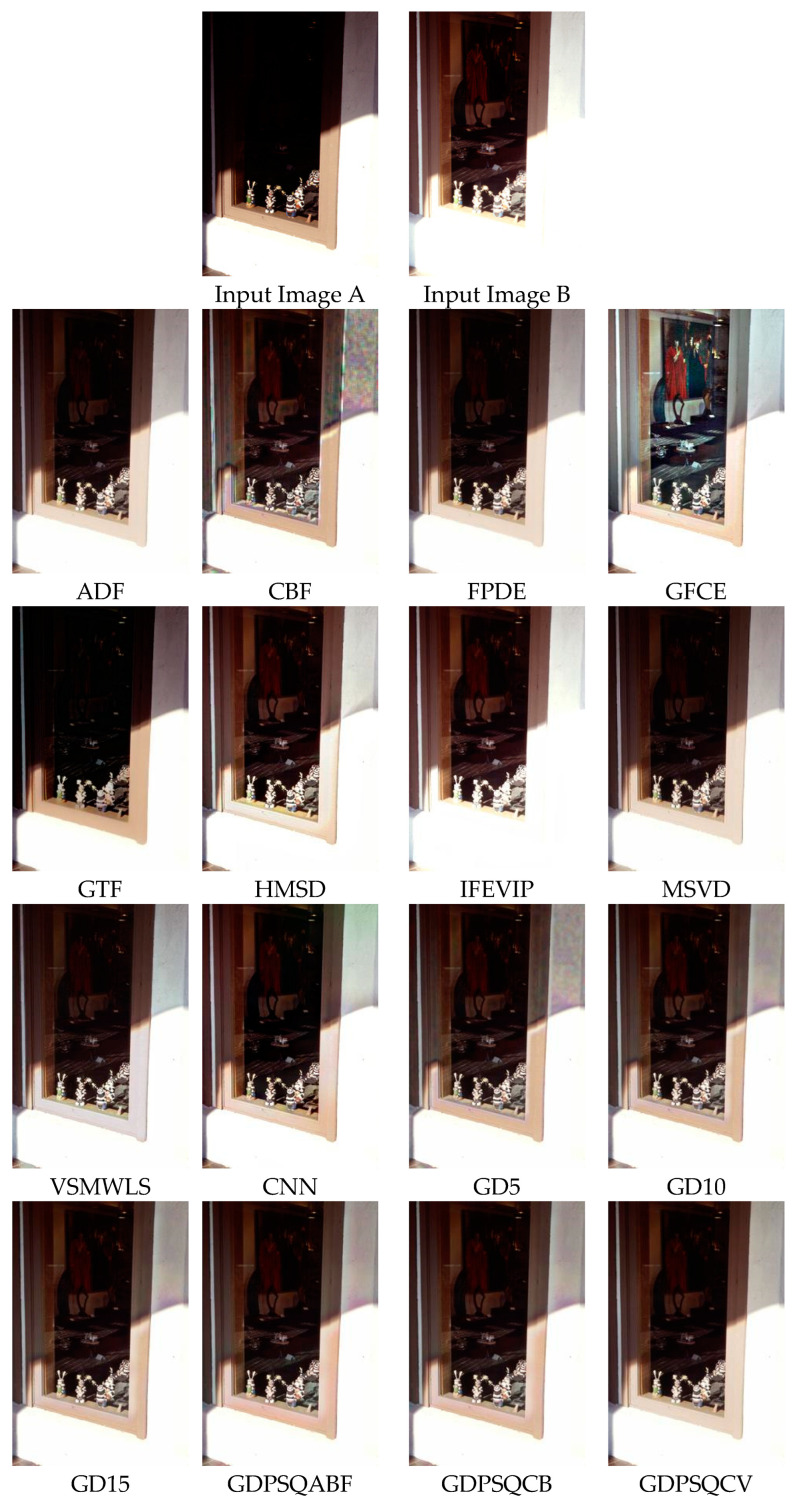
Multi-exposure image set E#6 (Images A and B) and their fusion image results, obtained using comparison methods.

The results of image set E#6 are given in Figure 20. As can be seen from the results, CBF, GTF, and GD5 show poor visual performance. Otherwise, GFCE, VSMWLS, HMSD, ADF, CNN, and the proposed GDPSQCV method show better visual performance than the other techniques.

In Table 11, the quantitative results of the comparison methods are given for image set E#6. As can be seen in the table, ADF, FPDE, and GDPSQCV show better performance according to the numerical results. On the other hand, GFCE, IFEVIP, and GTF show the worst performance compared to the others.

### 3.8. Overall Comparison

To evaluate the numerical results more easily, the average rankings of the methods with regards to all of the quality metrics were calculated for all 48 images used in the experiments. The best ranking was set to first, and the worst ranking was set to sixteenth according to the quality metric value of each method, as we have sixteen methods in total. Each fusion application type is given in a separate table.

Table 12 shows the ranking of each method for the fusion of multi-modal medical images, including M#1 to M#8. At the bottom of the table, the average ranking of each method compared to all of the images for medical image fusion is indicated. As can be seen in Table 12, overall better results in average ranking were obtained with GD10, GD15, and GDPSQCB, whose average ranking was around sixth. GFCE and MSVD were the two worst methods with an average ranking of ~12th.

Table 13 shows the ranking of each method for the fusion of infrared and visible images, including IV#1 to IV#14. As can be seen in Table 13, overall better average rankings were obtained with HMSD, GDPSQCV, GDPSQABF, and CNN, whose average ranking was around seventh. ~GTF was the worst method an average ranking of ~11th.

The ranking of each method for the fusion of multi-focus images, including F#1 to F#20, are given in Table 14. As can be seen from the results, overall better average rankings were obtained with GD15, GDPSQCV, GD10, CBF, and CNN, whose average ranking was around sixth. GFCE and IFEVIP were the worst methods with an average ranking of ~14th average ranking.

The ranking of each method for the fusion of multi-exposure images, including E#1 to E#6, are given in Table 15. As can be seen from the results, overall better average rankings were obtained with GDPSQCV, GDPSQABF, and ADF, whose average ranking was around fifth. GFCE was the worst method with an average ranking of ~13th.

The global average rankings and average CPU time consumptions of the methods for all 48 images are given in Table 16. As can be seen from the table, the proposed GD methods take the first three best rankings. The methods can be ordered from best to worst as GDPSQCV, GD15, GDPSQABF, GDPSQCB, GD10, HMSD, CNN, VSMWLS, ADF, FPDE, GD5, CBF, MSVD, GTF, IFEVIP, and GFCE. Table 16 also shows the global average CPU time consumptions of the methods in seconds. The execution time of an image processing method is directly affected by its complexity and the CPU capacity it is run on, as shown in [69]: the lower the CPU time, the faster the execution time of the method. According to the numerical results, IFEVIP, GD5, and GD10 are the fastest methods compared to the others.

## 4. Conclusions

In this paper, a general image fusion method based on the GD, linear weighting, and PS optimization is proposed. The main advantages of the proposed GD method can be summarized as follows:

- It is based on basic image convolution and linear weighting. Thus, the main algorithm is very simple and can be implemented on embedded systems and PCs and easily parallelized on multiple CPU or GPU cores.
- It is a pixel-based image fusion method, and the method does not utilize an image transform. Moreover, it does not require a training phase. Therefore, the proposed method is pretty fast compared to state-of-the-art fusion methods.
- The method relies on transferring information from each input image by enhancing the high-frequency components using simple, first-order derivative edge detection. Neighboring pixels also contribute to the center pixel’s weighting, proportional to their distance, using a Gaussian filter.
- The method has only two control parameters. In this paper, we define some predefined parameter sets and explore their performance. And a simple optimal solution to determine the adaptively control parameters is also proposed and compared.
- It can be used in any kind of image fusion application, such as multi-modal medical image fusion, infrared and visible image fusion for enhanced night vision, multi-focus image fusion for extending the depth of field, and multi-exposure image fusion for high-dynamic-range imaging.
- It can fuse more than two input images with the help of its generalized structure. Therefore, it can be used in future studies to fuse multi-spectral and hyperspectral images with 10–200 input images corresponding to different wavelengths in the visible and non-visible spectrum.

The proposed GD method with its six different versions has been compared with 10 state-of-the-art image fusion methods by utilizing qualitative and quantitative evaluation. In total, 48 pairs of test images were used in the experiments. However, only two pairs of test images were detailed and visualized for each of the four different types of image fusion in the experiments. The fusion results of all images in the dataset can be found at the Supplementary Materials section. In addition to visual subjective evaluations, nine objective quality metrics were utilized to compare the proposed GD method with other fusion methods.

Extensive experiments have shown that the proposed GDPSQCV method attained an average rank of 6.44th among 16 methods, when considering all quality metrics and all test images, which is the best ranking of all of the methods. Moreover, the average CPU consumption time of GD15, which is the second best in overall ranking, is about 0.20 s, which is only 0.05 s slower than IFEVIP (revealed as the fastest method in the experiments). However, it must be noted that IFEVIPs average ranking is 11.41th. In addition to this, the proposed GD15 is ~115× faster than the CNN method in terms of average CPU consumption time for the fusion of 48 image pairs on an Intel i7 CPU clocked @ 4 GHz PC without parallel programming. Increasing the Gaussian filter size increases the success of the proposed method. Namely, GD15 obtained better results than GD10, and GD10 obtained better results than GD5. However, unreported experiments showed that increasing the filter size causes undesirable visual effects on the fused image. Optimal versions of GD have better performance compared to their non-adaptive versions such as GD5, GD10, and GD15. However, the CPU computing times of GDPS versions are much higher.

The main limitation of the proposed method is that it does not guarantee the best result in a particular application. However, it is capable of being a general fusion scheme and gives better results in average for any kind of fusion application. In future studies, optimization algorithm and the fitness function to be optimized may be improved. Meta-heuristic algorithms are very promising, and multi-objective versions can improve the overall performance by optimizing two or more quality metrics together. In addition to this, GPU computing techniques may be utilized to speed up the optimization process. As a result, although it may not achieve the overall best result in all tests, the proposed GD method can be used as a simple and effective general image fusion method.

## Figures and Tables

**Table 1:** Specifications of the image dataset used in the experiments.

Application	Images in Dataset	Image Type	Resolution
Multi-modal medical	8	Graylevel TIF	256 × 256
Multi-sensor infrared and visible	14	Graylevel PNG	360 × 270, 430 × 340, 512 × 512, 632 × 496
Multi-focus	20	RGB JPG	520 × 520
Multi-exposure	6	RGB JPG	340 × 230, 230 × 340, 752 × 500
*Total*	*48*	*-*	*-*

**Table 2:** Specifications of the implemented environment for experiments.

Environmental Feature	Description
Operating system	Windows 10 Pro
CPU	Intel i7-4790K @ 4 GHz
GPU	Nvidia GeForce GTX 760
RAM	16 GB
Programming language	MATLAB 2023a

**Table 3:** Configuration parameters of the fusion methods used in the experiments.

Fusion Method	Configuration Parameters
ADF	num_iter = 10, delta_t = 0.15, kappa = 30, option = 1
CBF	cov_wsize = 5, sigmas = 1.8, sigmar = 25, ksize = 11
FPDE	n = 15, dt = 0.9, k = 4
GFCE	nLevel = 4, sigma = 2, k = 2, r0 = 2, eps0 = 0.1, l = 2
GTF	adapt_epsR = 1, epsR_cutoff = 0.01, adapt_epsF = 1, epsF_cutoff = 0.05, pcgtol_ini = 1 × 10^−4^, loops = 5, pcgtol_ini = 1 × 10^−2^, adaptPCGtol = 1,
HMSD	nLevel = 4, lambda = 30, sigma = 2.0, sigma_r = 0.05, k = 2,
IFEVIP	QuadNormDim = 512, QuadMinDim = 32, GaussScale = 9, MaxRatio = 0.001, StdRatio = 0.8,
MSVD	-
VSMWLS	sigma_s = 2, sigma_r = 0.05
CNN	type = siamese network, weights_b1_1 = 9 ∗ 64, weights_b1_2 = 64 ∗ 9 ∗ 128, weights_b1_3 = 128 ∗ 9 ∗ 256, weights_output= 512 ∗ 64 ∗ 2
Proposed GD5	*s* = 5, *σ* = 1.6
Proposed GD10	*s* = 10, *σ* = 3.3
Proposed GD15	*s* = 15, *σ* = 5
Proposed GDPSQABF	optimizer = pattern search, algorithm = classic, init_sol = [10; 3.3], lb = [5; 1], ub = [80; 100], max_iter = 20, fit_fun = −1 ∗ Qabf
Proposed GDPSQCB	optimizer = pattern search, algorithm = classic, init_sol = [10; 3.3], lb = [5; 1], ub = [80; 100], max_iter = 20, fit_fun = −1 ∗ Qcb
Proposed GDPSQCV	optimizer = pattern search, algorithm = classic, init_sol = [10; 3.3], lb = [5; 1], ub = [80; 100], max_iter = 20, fit_fun = Qcv

**Table 4:** Quality metric scores of medical images set M#2, obtained using comparison methods.

	EN	MI	PSNR	Qabf	SSIM	Q_cb_	CE	RMSE	Q_cv_
**ADF**	4.783	2.308	59.298	0.467	1.498	0.363	1.281	0.076	858.898
**CBF**	5.015	**2.494**	58.979	0.531	1.496	0.407	1.198	0.082	858.355
**FPDE**	4.836	2.339	**59.397**	0.433	1.505	0.348	1.190	**0.075**	840.941
**GFCE**	**7.615**	2.190	53.849	0.474	0.463	0.389	4.502	0.268	1643.875
**GTF**	4.813	2.248	58.770	**0.574**	1.486	**0.637**	**0.831**	0.086	1154.964
**HMSD**	4.831	2.286	58.628	**0.550**	1.488	0.442	0.852	0.089	999.258
**IFEVIP**	**5.153**	2.457	57.528	0.484	1.495	0.365	1.352	0.115	1242.540
**MSVD**	4.823	2.368	57.327	0.471	0.690	0.201	5.933	0.120	813.834
**VSMWLS**	**5.024**	2.352	59.033	0.529	**1.530**	**0.469**	**0.667**	0.081	964.498
**CNN**	4.932	2.337	58.484	**0.554**	1.505	**0.603**	**0.705**	0.092	1016.499
**GD5**	4.901	**2.478**	59.209	0.506	1.519	0.389	1.247	0.078	**805.711**
**GD10**	4.854	2.463	59.300	0.479	1.522	0.393	1.220	0.076	780.445
**GD15**	4.819	2.452	**59.342**	0.464	**1.522**	0.391	1.208	**0.076**	**773.312**
**GDPSQABF**	4.934	2.471	59.145	0.516	1.514	0.386	1.236	0.079	841.810
**GDPSQCB**	4.862	**2.472**	59.280	0.485	1.521	0.390	1.240	0.077	785.127
**GDPSQCV**	4.796	2.443	**59.369**	0.456	**1.524**	0.392	1.145	**0.075**	**781.420**

**Table 5:** Quality metric scores of medical images set M#5, obtained using comparison methods.

	EN	MI	PSNR	Qabf	SSIM	Q_cb_	CE	RMSE	Q_cv_
**ADF**	5.975	2.288	56.459	0.408	1.170	0.503	0.329	0.147	845.674
**CBF**	5.962	2.571	56.592	0.512	1.289	0.511	**0.293**	0.143	523.914
**FPDE**	**6.408**	2.122	56.007	0.305	1.019	0.475	0.404	0.163	896.311
**GFCE**	**7.311**	2.382	54.953	0.447	0.964	0.492	2.920	0.208	751.841
**GTF**	6.006	2.386	55.932	0.404	1.275	0.431	**0.287**	0.166	1677.168
**HMSD**	6.400	2.435	56.376	**0.513**	1.324	**0.519**	0.564	0.150	549.287
**IFEVIP**	6.348	2.554	55.266	**0.528**	1.338	0.508	0.798	0.193	628.882
**MSVD**	5.752	2.405	56.837	0.404	1.183	0.386	3.935	0.135	694.471
**VSMWLS**	6.170	**2.659**	56.588	0.512	1.355	**0.524**	0.344	0.143	495.160
**CNN**	**6.913**	**2.585**	56.001	**0.571**	1.277	**0.533**	1.427	0.163	**449.774**
**GD5**	5.822	2.557	56.906	0.473	1.352	0.467	0.329	0.133	500.209
**GD10**	5.791	**2.574**	**56.966**	0.453	**1.371**	0.479	0.338	**0.131**	**454.096**
**GD15**	5.776	2.562	**56.997**	0.436	**1.374**	0.470	0.340	**0.130**	454.362
**GDPSQABF**	5.820	2.553	56.901	0.474	1.349	0.483	**0.327**	0.133	511.725
**GDPSQCB**	5.796	2.564	56.954	0.457	1.367	0.481	0.331	0.131	455.932
**GDPSQCV**	5.782	2.568	**56.983**	0.444	**1.373**	0.474	0.339	**0.130**	**452.181**

**Table 6:** Quality metric scores of infrared and visible images set IV#4, obtained using comparison methods.

	EN	MI	PSNR	Qabf	SSIM	Q_cb_	CE	RMSE	Q_cv_
**ADF**	6.132	1.468	**60.947**	0.470	1.045	0.434	0.790	**0.052**	**118.387**
**CBF**	**6.730**	**1.919**	59.715	**0.632**	1.102	**0.473**	0.782	0.069	211.646
**FPDE**	6.159	1.325	**60.930**	0.481	1.027	0.434	0.740	**0.052**	**119.226**
**GFCE**	**7.644**	1.231	55.427	0.391	0.465	0.393	2.065	0.186	646.551
**GTF**	6.161	1.016	60.122	0.311	0.863	0.310	0.575	0.063	160.482
**HMSD**	6.070	1.485	60.356	0.579	0.998	0.462	**0.391**	0.060	165.976
**IFEVIP**	**6.869**	**2.143**	59.503	**0.670**	1.129	0.469	0.891	0.073	206.815
**MSVD**	6.024	1.578	**60.779**	0.309	0.944	0.339	5.285	**0.054**	154.207
**VSMWLS**	6.297	1.403	60.621	0.617	1.072	0.437	**0.494**	0.056	145.077
**CNN**	5.735	1.350	60.244	0.562	0.956	0.424	**0.282**	0.061	178.710
**GD5**	6.672	**1.791**	60.006	0.628	1.135	0.469	0.812	0.065	152.836
**GD10**	6.670	1.761	60.027	**0.632**	**1.148**	**0.470**	0.798	0.065	146.837
**GD15**	6.665	1.723	60.052	0.629	**1.151**	0.469	0.787	0.064	135.276
**GDPSQABF**	6.671	1.769	60.023	0.632	**1.147**	0.469	0.802	0.065	148.808
**GDPSQCB**	6.672	1.763	60.029	0.630	1.146	**0.470**	0.796	0.065	145.011
**GDPSQCV**	6.495	1.479	60.470	0.564	1.127	0.463	0.763	0.058	**78.475**

**Table 7:** Quality metric scores of infrared and visible images set IV#5, obtained using comparison methods.

	EN	MI	PSNR	Qabf	SSIM	Q_cb_	CE	RMSE	Q_cv_
**ADF**	5.981	2.091	**58.438**	0.588	**1.422**	0.415	3.677	**0.093**	649.629
**CBF**	**6.896**	**2.822**	57.178	0.600	1.227	0.492	2.157	0.125	639.983
**FPDE**	5.972	2.149	**58.439**	0.559	**1.422**	0.418	3.275	**0.093**	625.309
**GFCE**	**7.230**	2.124	57.075	0.558	1.327	0.375	3.706	0.128	**80.675**
**GTF**	5.520	1.997	58.210	0.183	1.380	0.323	2.877	0.098	2764.969
**HMSD**	6.722	2.092	58.250	0.613	1.412	0.368	1.318	0.097	**237.620**
**IFEVIP**	6.409	**3.898**	57.700	0.551	1.362	0.366	**0.918**	0.110	**246.528**
**MSVD**	**6.870**	**2.735**	58.210	0.625	1.334	0.459	3.007	0.098	609.813
**VSMWLS**	6.129	1.800	**58.400**	0.647	1.408	0.403	5.896	**0.094**	667.889
**CNN**	6.781	2.059	57.686	**0.743**	1.345	0.411	4.080	0.111	256.092
**GD5**	6.738	2.334	57.497	0.567	1.285	0.497	3.769	0.116	524.444
**GD10**	6.693	2.349	57.528	0.625	1.351	0.519	3.983	0.115	503.300
**GD15**	6.677	2.303	57.559	**0.652**	1.370	**0.541**	**1.177**	0.114	496.323
**GDPSQABF**	6.647	2.169	57.616	**0.658**	1.383	**0.543**	**1.307**	0.113	492.907
**GDPSQCB**	6.395	1.502	57.954	0.618	**1.421**	0.535	1.852	0.104	729.452
**GDPSQCV**	6.677	2.312	57.546	0.630	1.360	**0.536**	4.001	0.114	487.171

**Table 8:** Quality metric scores of multi-focus images set F#11, obtained using comparison methods.

	EN	MI	PSNR	Qabf	SSIM	Q_cb_	CE	RMSE	Q_cv_
**ADF**	7.669	4.513	**63.818**	0.610	1.654	0.643	**0.017**	**0.027**	101.099
**CBF**	7.681	**5.319**	63.383	**0.752**	1.647	**0.758**	0.019	0.030	**20.292**
**FPDE**	7.661	4.401	**63.914**	0.570	1.663	0.622	0.021	**0.026**	91.789
**GFCE**	6.962	2.861	58.590	0.600	1.419	0.527	0.543	0.090	130.958
**GTF**	7.670	4.585	63.464	0.708	1.637	0.660	**0.019**	0.029	65.129
**HMSD**	7.650	**4.999**	63.173	**0.738**	1.642	**0.742**	0.020	0.031	**15.926**
**IFEVIP**	7.019	2.661	59.720	0.449	1.500	0.505	0.361	0.069	321.098
**MSVD**	7.669	4.149	63.421	0.427	1.633	0.616	0.020	0.030	94.007
**VSMWLS**	7.666	4.424	63.498	0.674	1.655	0.664	**0.015**	0.029	39.009
**CNN**	7.668	**5.404**	63.106	**0.757**	1.635	**0.769**	0.030	0.032	**14.200**
**GD5**	**7.688**	4.754	63.655	0.724	1.665	0.710	0.023	0.028	32.352
**GD10**	7.685	4.747	63.696	0.723	**1.667**	0.712	0.022	0.028	27.732
**GD15**	7.684	4.745	63.714	0.722	**1.667**	0.713	0.022	0.028	26.936
**GDPSQABF**	**7.688**	4.756	63.651	0.725	1.665	0.709	0.023	0.028	33.095
**GDPSQCB**	**7.685**	4.747	63.696	0.722	1.667	0.712	0.022	0.028	27.694
**GDPSQCV**	7.683	4.709	**63.781**	0.714	**1.669**	0.707	0.022	**0.027**	26.191

**Table 9:** Quality metric scores of multi-focus images set F#15, obtained using comparison methods.

	EN	MI	PSNR	Qabf	SSIM	Q_cb_	CE	RMSE	Q_cv_
**ADF**	7.611	5.753	**68.864**	0.748	**1.856**	0.755	**0.009**	**0.008**	3.640
**CBF**	7.628	**6.445**	68.394	**0.805**	1.840	**0.815**	0.011	0.009	3.873
**FPDE**	7.614	5.617	**68.806**	0.744	**1.854**	0.725	0.013	**0.009**	3.734
**GFCE**	**7.636**	3.140	57.958	0.610	1.396	0.625	0.971	0.105	94.969
**GTF**	7.623	**6.540**	**69.036**	**0.791**	1.837	0.786	0.011	**0.008**	5.307
**HMSD**	**7.628**	5.958	68.060	0.789	1.836	0.779	0.012	0.010	4.031
**IFEVIP**	**7.632**	3.663	60.891	0.627	1.674	0.616	0.321	0.053	158.223
**MSVD**	7.579	4.972	66.507	0.520	1.784	0.711	**0.010**	0.015	6.843
**VSMWLS**	7.626	5.828	68.217	0.787	1.838	0.751	0.012	0.010	3.528
**CNN**	7.626	**6.829**	68.088	**0.811**	1.837	**0.829**	0.011	0.010	3.618
**GD5**	7.624	5.941	68.613	0.789	1.847	0.784	0.010	0.009	**3.195**
**GD10**	7.623	5.940	68.629	0.787	1.848	0.786	0.010	0.009	3.211
**GD15**	7.623	5.937	68.636	0.787	**1.848**	**0.787**	**0.010**	0.009	3.225
**GDPSQABF**	7.624	5.938	68.609	0.789	1.847	0.783	0.010	0.009	**3.194**
**GDPSQCB**	7.624	5.940	68.617	0.789	1.847	0.785	0.010	0.009	**3.194**
**GDPSQCV**	7.624	5.939	68.613	0.789	1.847	0.784	0.010	0.009	3.207

**Table 10:** Quality metric scores of multi-exposure images set E#5, obtained using comparison methods.

	EN	MI	PSNR	Qabf	SSIM	Q_cb_	CE	RMSE	Q_cv_
**ADF**	6.530	**3.440**	**58.730**	0.700	**1.719**	0.578	0.544	**0.087**	**69.401**
**CBF**	**6.704**	3.064	58.370	0.674	1.641	0.593	0.537	0.095	99.078
**FPDE**	6.498	**3.433**	**58.732**	0.697	**1.720**	0.576	0.547	**0.087**	**69.466**
**GFCE**	5.133	2.764	57.641	0.569	1.607	0.469	1.615	0.112	165.118
**GTF**	6.027	2.950	58.222	0.638	1.670	0.509	0.592	0.098	112.981
**HMSD**	**6.683**	3.317	58.387	0.703	1.656	**0.675**	0.669	0.094	98.335
**IFEVIP**	5.534	2.471	57.822	0.551	1.601	0.477	0.993	0.108	188.610
**MSVD**	6.524	3.329	58.690	0.691	1.701	0.582	0.555	0.088	70.008
**VSMWLS**	6.541	3.278	58.663	0.703	1.700	0.607	0.593	0.089	74.676
**CNN**	6.539	2.893	58.400	0.702	1.690	0.618	1.241	0.094	92.188
**GD5**	**6.676**	3.342	58.618	0.713	1.693	0.600	**0.532**	0.089	76.043
**GD10**	6.665	3.334	58.636	**0.716**	1.699	0.617	**0.536**	0.089	73.182
**GD15**	6.655	3.328	58.647	**0.716**	1.703	**0.622**	0.539	0.089	72.057
**GDPSQABF**	6.643	3.349	58.676	0.714	1.708	0.617	0.547	0.088	71.193
**GDPSQCB**	6.655	3.316	58.647	**0.715**	1.702	**0.624**	0.539	0.089	72.086
**GDPSQCV**	6.606	**3.439**	**58.716**	0.707	**1.716**	0.608	**0.533**	**0.087**	**68.797**

**Table 11:** Quality metric scores of multi-exposure images set E#6, obtained using comparison methods.

	EN	MI	PSNR	Qabf	SSIM	Q_cb_	CE	RMSE	Q_cv_
**ADF**	6.382	**3.912**	**57.541**	0.660	**1.510**	0.520	**0.792**	**0.115**	**88.447**
**CBF**	**6.674**	3.308	56.844	0.680	1.377	0.550	0.881	0.135	168.641
**FPDE**	6.381	**3.904**	**57.541**	0.659	**1.509**	0.523	0.868	**0.115**	**88.193**
**GFCE**	**6.749**	2.497	54.457	0.644	1.123	0.510	3.677	0.233	241.984
**GTF**	5.664	3.065	57.035	0.594	1.431	0.555	**0.609**	0.129	201.843
**HMSD**	**6.661**	3.289	57.130	0.691	1.461	0.521	1.065	0.126	132.652
**IFEVIP**	6.100	3.716	57.112	0.619	1.458	0.468	1.409	0.126	126.541
**MSVD**	6.385	**3.829**	**57.518**	0.637	1.498	0.521	**0.800**	**0.115**	89.599
**VSMWLS**	6.469	3.650	57.467	0.669	1.477	0.540	0.899	0.117	**88.157**
**CNN**	6.372	3.141	57.094	0.704	1.449	0.538	1.912	0.127	125.477
**GD5**	6.597	3.442	57.200	0.709	1.452	0.550	0.902	0.124	119.147
**GD10**	6.608	3.489	57.232	**0.716**	1.475	**0.567**	0.924	0.123	114.830
**GD15**	6.613	3.492	57.263	**0.716**	1.485	**0.570**	0.829	0.122	111.714
**GDPSQABF**	6.616	3.486	57.294	0.715	1.490	0.566	0.851	0.121	108.474
**GDPSQCB**	6.619	3.488	57.277	**0.717**	1.488	**0.570**	0.837	0.122	110.358
**GDPSQCV**	6.487	3.619	57.466	0.687	**1.510**	0.536	0.903	0.117	95.170

**Table 12:** Average rankings of the methods with regard to their quality metrics for multi-modal medical images.

	ADF	CBF	FPDE	GFCE	GTF	HMSD	IFEVIP	MSVD	VSMWLS	CNN	GD5	GD10	GD15	GDPSQABF	GDPSQCB	GDPSQCV
**Img. M#1 Rank.**	8.78	7.00	7.78	12.89	11.44	6.78	9.00	10.44	7.22	8.78	8.00	7.11	7.78	8.00	6.78	8.22
**Img. M#2 Rank.**	11.00	6.78	8.33	13.00	9.33	9.44	11.00	12.89	5.78	8.00	7.11	5.89	6.67	7.56	6.67	6.56
**Img. M#3 Rank.**	6.78	9.56	7.56	11.56	12.22	7.56	7.33	15.22	8.22	6.56	8.89	6.44	7.00	6.78	6.56	7.78
**Img. M#4 Rank.**	7.67	6.89	8.67	13.78	11.67	5.00	8.22	13.33	9.67	7.89	8.67	6.89	6.89	7.33	6.67	6.78
**Img. M#5 Rank.**	10.56	6.56	12.33	12.00	11.78	8.22	9.22	12.67	5.78	6.44	7.33	6.00	7.00	7.22	6.44	6.44
**Img. M#6 Rank.**	15.22	8.22	10.11	11.56	10.11	6.00	5.44	9.78	5.56	8.11	8.11	7.56	6.89	7.44	8.78	7.11
**Img. M#7 Rank.**	9.56	7.00	9.33	13.56	12.44	8.44	9.89	12.56	5.67	7.56	7.33	6.44	5.67	7.44	6.33	6.78
**Img. M#8 Rank.**	7.33	10.22	9.67	13.56	12.22	6.11	7.33	13.67	6.11	7.89	9.56	7.11	5.89	7.11	5.67	6.56
**Avg. Ranking**	9.61	7.78	9.22	12.74	11.40	7.19	8.43	12.57	6.75	7.65	8.13	**6.68**	**6.72**	7.36	**6.74**	7.03

**Table 13:** Average rankings of the methods with regard to their quality metrics for infrared and visible images.

Infrared and Visible Images	ADF	CBF	FPDE	GFCE	GTF	HMSD	IFEVIP	MSVD	VSMWLS	CNN	GD5	GD10	GD15	GDPSQABF	GDPSQCB	GDPSQCV
**Img. IV#1 Rank.**	5.67	11.33	7.11	11.11	10.00	5.56	10.33	10.11	7.22	5.67	10.33	8.67	8.44	9.11	7.44	7.89
**Img. IV#2 Rank.**	8.00	11.22	8.33	10.22	10.56	5.78	10.11	8.78	7.33	6.22	10.89	9.33	7.89	7.22	7.33	6.78
**Img. IV#3 Rank.**	6.11	11.67	7.11	10.44	10.89	5.33	11.00	10.00	6.44	5.67	10.11	8.56	8.00	9.00	8.11	7.56
**Img. IV#4 Rank.**	7.89	7.33	8.11	13.67	11.56	8.78	8.33	11.11	7.33	10.44	8.22	6.78	6.22	7.33	6.11	6.78
**Img. IV#5 Rank.**	8.33	9.44	7.78	10.67	11.33	6.44	7.89	7.11	9.44	8.67	10.22	8.89	6.67	6.22	8.56	8.33
**Img. IV#6 Rank.**	8.78	9.89	7.22	8.22	9.78	6.33	9.33	9.00	7.56	9.78	10.00	8.56	6.89	6.67	11.22	6.78
**Img. IV#7 Rank.**	7.11	11.56	7.44	9.00	12.22	5.22	9.22	8.67	9.89	5.00	11.56	10.00	8.56	6.00	9.11	5.44
**Img. IV#8 Rank.**	8.67	10.56	10.78	10.89	10.78	6.22	8.44	10.11	6.78	6.33	10.00	9.00	8.00	6.67	6.56	6.22
**Img. IV#9 Rank.**	7.89	10.11	7.11	11.22	15.00	7.33	10.67	7.89	5.78	6.44	10.56	7.67	7.00	7.00	6.67	7.67
**Img. IV#10 Rank.**	7.56	11.22	8.33	8.78	9.33	8.56	9.22	11.78	7.00	7.56	11.22	9.56	8.00	5.33	6.22	6.33
**Img. IV#11 Rank.**	8.11	11.33	8.11	12.78	7.56	9.67	6.78	6.67	5.33	6.78	10.44	9.78	8.33	6.11	11.33	6.89
**Img. IV#12 Rank.**	10.44	8.33	10.11	12.00	12.11	7.11	10.11	9.22	9.89	7.22	9.44	6.89	6.00	6.56	5.56	5.00
**Img. IV#13 Rank.**	7.78	12.33	7.89	7.89	13.00	7.11	5.89	9.00	6.44	6.44	10.11	8.78	7.89	7.56	10.89	7.00
**Img. IV#14 Rank.**	8.11	12.11	8.11	7.89	11.22	5.67	5.33	9.89	6.22	7.22	10.11	8.89	8.67	7.89	10.67	8.00
**Avg. Ranking**	7.89	10.60	8.11	10.34	11.10	**6.79**	8.76	9.24	7.33	7.10	10.23	8.67	7.61	**7.05**	8.27	**6.91**

**Table 14:** Average rankings of the methods with regard to their quality metrics for multi-focus images.

Multi-Focus Images	ADF	CBF	FPDE	GFCE	GTF	HMSD	IFEVIP	MSVD	VSMWLS	CNN	GD5	GD10	GD15	GDPSQABF	GDPSQCB	GDPSQCV
**Img. F#1 Rank.**	9.44	5.67	8.89	11.67	9.89	6.89	14.11	12.89	8.11	6.22	7.56	6.89	7.44	6.67	6.89	6.78
**Img. F#2 Rank.**	7.78	6.89	9.78	13.56	9.33	7.44	13.89	9.56	7.89	6.78	7.11	7.22	6.44	7.00	7.56	7.78
**Img. F#3 Rank.**	9.44	6.56	9.78	13.78	10.89	7.00	13.44	9.56	7.89	7.89	7.67	6.67	5.89	7.00	6.33	6.22
**Img. F#4 Rank.**	8.11	5.78	9.22	15.44	11.78	10.11	13.67	10.56	7.44	7.11	7.11	5.67	5.56	6.33	6.11	6.00
**Img. F#5 Rank.**	7.11	6.33	10.00	14.89	9.67	7.67	15.89	9.56	9.33	7.00	6.44	5.78	6.22	7.44	6.67	6.00
**Img. F#6 Rank.**	7.67	6.89	9.78	13.78	9.56	8.00	13.44	9.00	8.67	8.11	7.44	6.78	6.44	7.22	6.56	6.67
**Img. F#7 Rank.**	9.11	6.78	9.44	15.22	10.56	7.44	14.22	8.67	7.33	7.00	7.56	6.22	5.67	8.44	7.11	5.22
**Img. F#8 Rank.**	8.89	6.22	9.44	14.00	7.33	9.56	13.78	12.44	8.44	6.33	6.67	7.22	5.89	6.89	6.89	6.00
**Img. F#9 Rank.**	9.00	6.78	9.89	12.67	9.89	7.11	13.44	8.89	9.33	8.22	8.00	6.78	6.33	7.00	6.11	6.56
**Img. F#10 Rank.**	8.11	6.78	9.22	14.22	10.11	6.89	13.44	13.22	6.67	6.89	7.22	6.67	6.00	7.56	6.44	6.56
**Img. F#11 Rank.**	8.22	6.00	9.00	15.33	9.44	7.44	15.33	12.00	9.11	7.78	6.56	5.78	5.67	6.67	5.89	5.78
**Img. F#12 Rank.**	8.00	6.11	8.67	15.33	9.78	8.33	13.78	10.22	8.78	7.89	7.89	6.11	5.44	7.56	6.33	5.78
**Img. F#13 Rank.**	8.67	7.56	9.89	13.67	9.00	8.11	13.78	10.22	9.22	8.22	7.56	6.11	5.56	6.00	6.78	5.67
**Img. F#14 Rank.**	9.00	6.22	9.67	15.33	8.78	6.89	14.67	10.44	9.11	7.56	7.22	6.33	6.33	6.56	6.11	5.78
**Img. F#15 Rank.**	7.22	6.78	9.44	14.00	6.56	9.44	13.67	13.22	10.00	6.67	6.56	6.33	6.22	7.11	5.78	7.00
**Img. F#16 Rank.**	7.22	7.89	8.33	15.22	9.67	9.89	15.44	8.44	7.44	9.44	7.11	5.44	5.78	6.67	6.11	5.89
**Img. F#17 Rank.**	9.11	6.67	9.56	14.44	9.89	7.22	15.56	9.56	9.22	6.78	7.78	5.67	6.22	6.44	5.78	6.11
**Img. F#18 Rank.**	7.78	7.78	9.56	14.33	9.33	10.11	13.56	8.22	7.33	7.11	6.67	6.67	6.56	8.11	6.56	6.33
**Img. F#19 Rank.**	8.67	6.22	9.67	14.11	9.89	7.11	15.44	10.78	9.11	7.22	7.67	6.22	5.00	6.56	5.56	6.78
**Img. F#20 Rank.**	8.89	5.67	10.22	15.33	10.22	7.11	13.78	8.67	7.67	6.67	7.67	6.33	6.67	7.11	6.67	7.33
**Avg. Ranking**	8.37	6.58	9.47	14.32	9.58	7.99	14.22	10.31	8.40	7.34	7.27	6.34	6.07	7.02	6.41	6.31

**Table 15:** Average rankings of the methods with regard to their quality metrics for multi-exposure images.

Multi-Exposure Images	ADF	CBF	FPDE	GFCE	GTF	HMSD	IFEVIP	MSVD	VSMWLS	CNN	GD5	GD10	GD15	GDPSQABF	GDPSQCB	GDPSQCV
**Img. E#1 Rank.**	6.11	14.00	5.78	13.78	12.44	7.44	10.56	8.00	7.22	7.89	11.11	8.56	6.78	4.78	6.33	5.22
**Img. E#2 Rank.**	5.33	10.44	5.44	13.22	11.56	10.78	15.89	7.67	6.56	9.56	8.67	7.67	6.89	5.33	6.33	4.67
**Img. E#3 Rank.**	6.33	13.22	6.78	11.11	10.44	8.33	9.89	6.00	7.22	10.33	11.67	8.89	8.11	5.56	7.33	4.78
**Img. E#4 Rank.**	5.00	13.67	5.11	13.56	11.22	6.11	9.11	7.11	9.00	7.56	12.33	9.89	8.33	6.33	6.67	5.00
**Img. E#5 Rank.**	5.44	10.33	6.00	15.56	13.33	9.00	15.33	7.89	8.56	10.67	7.00	6.00	5.33	5.44	6.22	3.89
**Img. E#6 Rank.**	5.44	10.78	5.78	13.89	12.22	10.44	12.33	6.33	6.56	12.00	9.11	7.67	5.67	5.78	5.33	6.67
**Avg. Ranking**	**5.61**	12.07	5.82	13.52	11.87	8.68	12.19	7.17	7.52	9.67	9.98	8.11	6.85	**5.54**	6.37	**5.04**

**Table 16:** Global average rankings of the methods with regard to their quality metrics and average CPU time consumptions (s) for all images.

All Images	ADF	CBF	FPDE	GFCE	GTF	HMSD	IFEVIP	MSVD	VSMWLS	CNN	GD5	GD10	GD15	GDPSQABF	GDPSQCB	GDPSQCV
**Avg. Ranking**	8.09	8.64	8.58	12.79	10.61	7.59	11.41	9.98	7.71	7.62	8.62	7.30	**6.72**	**6.90**	7.00	**6.44**
**Avg. CPU Time**	0.56	14.08	1.76	1.46	5.63	6.28	**0.15**	0.57	2.30	22.99	**0.16**	**0.18**	0.20	19.65	15.40	21.72

## Data Availability

Publicly available datasets were analyzed in this study. These data and MATLAB codes of the proposed GD method will be released at: https://github.com/rifatkurban/GDfusion.

## Supplementary Materials

The following supporting information can be downloaded at: https://github.com/rifatkurban/GDfusion, fused images and numerical results of input image pairs in the dataset.
